# Aerobic exercise training combined with local strength exercise restores muscle blood flow and maximal aerobic capacity in long-term Hodgkin lymphoma survivors

**DOI:** 10.1152/ajpheart.00132.2024

**Published:** 2024-04-19

**Authors:** Luciana De Souza Santos, Marília Harumi Higuchi dos Santos Rehder, Marcelo Vailati Negrao, Beatriz R. Goes-Santos, Edgar Toshi Dias, Camila Jordão Paixão, Ursula Urias, Natali Schiavo Giannetti, Ludhmila A. Hajjar, Roberto Kalil Filho, Carlos E. Negrão

**Affiliations:** ^1^Instituto do Coração, Hospital das Clínicas da Faculdade de Medicina da Universidade de São Paulo, Faculdade de Medicina, Universidade de São Paulo, São Paulo, Brazil; ^2^Escola de Educação Física e Esporte, Universidade de São Paulo, São Paulo, Brazil; ^3^Instituto do Câncer do Estado de São Paulo, Hospital das Clínicas HCFMUSP, Faculdade de Medicina, Universidade de São Paulo, São Paulo, Brazil; ^4^Department of Thoracic/Head and Neck Medical Oncology, The University of Texas MD Anderson Cancer Center, Houston, Texas, United States; ^5^School of Physical Education, University of Campinas, Campinas, Brazil; ^6^Methodist University of São Paulo, São Paulo, Brazil

**Keywords:** exercise training, forearm blood flow, Hodgkin lymphoma, maximal aerobic capacity

## Abstract

It is unclear whether muscle blood flow (MBF) is altered in long-term Hodgkin lymphoma (HL) survivors. We tested the hypothesis that *1*) MBF response during mental stress (MS) is impaired in long-term HL survivors and *2*) aerobic exercise training combined with local strength exercise (ET) restores MBF responses during MS in these survivors. Eighteen 5-year HL survivors and 10 aged-paired healthy subjects (HC) were studied. Twenty HL survivors were randomly divided into two groups: exercise-trained (HLT, *n* = 10) and untrained (HLUT, *n* = 10). Maximal aerobic capacity was evaluated by a cardiopulmonary exercise test and forearm blood flow (FBF) by venous occlusion plethysmography. MS was elicited by Stroop color and word test. ET was conducted for 4 mo, 3/wk for 60 min each session. The aerobic exercise intensity corresponded to anaerobic threshold up to 10% below the respiratory compensation point. The strength exercises consisted of two to three sets of chest press, pulley and squat exercises, 12–15 repetitions each exercise at 30–50% of the maximal voluntary contraction. Baseline was similar in HL survivors and HC, except peak oxygen consumption (peak V̇o_2_, *P* = 0.013) and FBF (*P* = 0.006) that were lower in the HL survivors. FBF responses during MS were lower in HL survivors (*P* < 0.001). ET increased peak V̇o_2_ (11.59 ± 3.07%, *P* = 0.002) and FBF at rest (33.74 ± 5.13%, *P* < 0.001) and during MS (24 ± 5.31%, *P* = 0.001). Further analysis showed correlation between the changes in peak V̇o_2_ and the changes in FBF during MS (r = 0.711, *P* = 0.001). In conclusion, long-term HL survivors have impaired MBF responses during MS. ET restores MBF responses during MS.

**NEW & NOTEWORTHY** Long-term Hodgkin lymphoma (HL) survivors have impaired muscle blood flow responses during mental stress and decreased maximal aerobic capacity. Supervised aerobic exercise training combined with local strength exercises restores muscle blood flow responses during mental stress and maximal aerobic capacity in these survivors. These findings provide evidence of safety and effectiveness of exercise training in HL survivors. Moreover, they highlight the importance of exercise training in the treatment of this set of patients.

## INTRODUCTION

Hodgkin lymphoma (HL) is the most common form of lymphoma (1). If diagnosed in the early stage, HL is highly curable, reaching a survival rate of 90–96% (1). Despite this remarkable result, many patients die prematurely because of cardiovascular disease (2). The incdence of cardiovascular mortality in patients in stages I and II of HL exceeds the incidence of HL and other types of neoplasia (3). The explanation for this scenario is not fully understood. However, there is evidence that drugs and radiation used as standard care in patients with HL play a role in the development of cardiovascular disease (4, 5).

Studies show that cardiac toxicity caused by chemotherapy and radiation can provoke myocardium systolic dysfunction and heart failure in a more advanced stage (6). There is also evidence that chemotherapy and radiation can affect the entire cardiovascular system. Coronary artery disease, cardiac diastolic dysfunction, alteration in cardiac conduction and arrhythmias, alteration in systemic and pulmonary vascular function, and thrombosis have been reported after treatment with chemotherapy and radiation (7). These side effects when associated with hypertension, weight gain, cigarette smoking, and physical inactivity aggravate fatigue and reduction in maximal aerobic capacity in patients with HL (8, 9). In the attempt to interrupt this vicious circle, patients with a diagnosis of HL minimal of 5 years were invited to participate in an exercise training (ET) program. The rational for choosing exercise training is that this nonpharmacological strategy substantially improves vascular function in patients with cardiovascular disease (10, 11). In addition, ET remarkably increases maximal aerobic capacity in patients with ischemic cardiovascular disease and heart failure (12).

We test the hypothesis that the muscle blood flow response during mental stress is impaired in long-term HL survivors when compared with healthy individuals. In addition, ET based on aerobic exercise and strengthen exercise restores the muscle blood flow responses during mental stress in these survivors.

## MATERIALS AND METHODS

### Study Population

Patients with anatomopathological diagnosis of HL minimal of 5 years (average 8 years) after being submitted to chemotherapy with anthracycline (ABVD protocol) and mediastinal radiotherapy, age >18 yr old, both sexes with preserved left ventricular ejection fraction (>50%) were selected for the study. The patients who were pregnant or had kidney insufficiency (creatinine > 1.5 mg/dL), iodine allergy, myocardial ischemia during cardiopulmonary exercise test, Eastern Cooperative Oncology Group scale (ECOG) = 3 or greater, and/or Karnofsky Performance Status (KPS) = 60 or lower, and life expectancy < 1 year were excluded. All women were under regular menstrual period. Ten age-paired healthy controls (HC) were also enrolled in the study. The study was approved by the Institutional Review Board of the Instituto do Coração (InCor-HCFMUSP) (SDC No. 4327/15/154) and by the Human Subject Protection Committee of the Hospital das Clínicas, Faculdade de Medicina, Universidade de São Paulo (HCFMUSP), São Paulo, SP, Brazil (CAAE No. 62084016.2.0000.0065). The trial is registered at www.ClinicalTrials.gov (No. NCT04636255). All experimental procedures and measurements were conducted according to the Declaration of Helsinki.

### Methods

Forearm blood flow (FBF) was assessed by venous occlusion plethysmography as described elsewhere (13). Briefly, a silastic band filled with mercury was placed around the forearm, connected to a plethysmograph device (Hokanson-AI-6). Two cuffs were placed in the dominant arm, one in the wrist and one in the forearm. The wrist cuff was inflated at 200 mmHg and maintained throughout the whole experimental protocol. Meanwhile, the forearm cuff was inflated at 60 mmHg for 7 to 8 s and deflated for an equal time, completing 15–16 s. FBF has been extensively used as an estimation of muscle blood flow in humans. During Stroop color and word test, the FBF measures are very useful to evaluate the muscle vascular function during a stressful condition. The reproducibility of FBF measured by venous occlusion plethysmography at different time intervals in the same individual in our laboratory is *r* = 0.93 (14).

Arterial blood pressure and pulse rate were obtained noninvasively on a beat-to-beat basis by finger photoplethysmography (FinometerPro; Finapress Medical Systems, Amsterdam, The Netherlands).

Transthoracic echocardiography was conducted to evaluate the cardiac function (Vivid E9, General Electric, Horten, Norway). The left ventricular end-diastolic and end-systolic volumes were assessed for calculation of the left ventricular ejection fraction (LVEF) by Simpson’s biplane method (15).

Maximal aerobic capacity was assessed by a cardiopulmonary exercise on a cycle ergometer (Medifit 400 L, Medical Fitness Equipment), using a ramp protocol with workload increment of 10–15 W/min, maintaining 60 rpm until exhaustion (16). The oxygen uptake (V̇o_2_) and the carbon dioxide production were assessed on a breath-by-breath basis on a computerized system (Vmax Encore 29 System; VIASYS Healthcare, Yorba Linda, CA). The completion of the test occurred when the patient could no longer maintain the exercise intensity and respiratory exchange ratio reached a value > 1.10. Heart rate was continuously recorded using a 12-lead digital electrocardiogram (ERGO PC 13, MICROMED Biotechnology).

ET was conducted for 4 mo, 3/wk under supervision. The exercise session consisted of 5-min stretching exercises, 40 min of cycling on a bicycle ergometer, 10 min of local strength exercises, and 5 min of cool down with stretching exercises. The relative aerobic exercise intensity was established by heart rate levels that corresponded to an anaerobic threshold up to 10% below the respiratory compensation point obtained in the cardiopulmonary exercise test, similar to the exercise training program that we have previously reported in patients with heart failure (16). The local strength exercise consisted of two to three sets of chest press, pulley, and squat exercises, 12–15 repetitions for each exercise at 30–50% of the maximal voluntary contraction performed on isokinetic dynamometer.

Mental stress was elicited by Stroop Color Word test (13). Briefly, a series of names of colors written in a different color ink from the color specified are presented to the subject. The individual needed to identify the color of the ink, not read the word. Stroop Color Word test is a very useful tool to examine the muscle vasodilation response during mental stress in humans. Moreover, this test differentiates muscle vasodilation response in patients with cardiovascular disease, COVID-19 survivors, and healthy individuals (13, 17, 18).

Arterial blood pressure, pulse rate, and FBF were recorded throughout the experimental protocol. The study was conducted at a room-controlled temperature (21–22°C) in the supine position. Caffeine and alcohol intake was not allowed for 24 h before the study. Cuffs for FBF measures were positioned in the dominant arm. A cuff with an appropriate size for the middle finger was placed in the nondominant hand for blood pressure and pulse rate measures. The investigators responsible for assessing outcome measures were not blinded to group assignments in the exercise intervention.

### Statistical Analysis

The sample size calculation was based on the previous work (19). The G*Power 3.1.9.7 statistical program based on a *t* test for two independent groups with an α error of 0.05 and a β error of 0.20 indicated that the sample was nine subjects in each group. Total area under the curve (AUC) of the FBF, arterial pressure, and pulse rate during mental stress was calculated using GraphPad Prism (v. 8.4). Data are shown as means ± SE or frequency with percentage. The normality was verified by Shapiro–Wilk test. Continuous variables were tested by unpaired independent *t* test or ANOVA. Two-way analysis of variance was used to test differences between groups. In case of significant differences, Bonferroni’s post hoc analysis was used. χ^2^ was used to compare categorical variables. Pearson correlation was used when appropriate. Statistical significance was set with a *P* < 0.05. Statistical Package for the Social Science 25.0 (SPSS, IBM) was used for all statistical analysis, and GraphPad Prism 8.4 was used for figures. The randomization to exercise-trained group and untrained group was conducted on a one-to-one ratio.

## RESULTS

### Impact of Hodgkin Lymphoma

#### Baseline measures.

The baseline characteristics are shown in Table 1. Sex, age, and body mass index (BMI) were not different between HL survivors and HC groups. Systolic, diastolic, and mean arterial pressure were also similar between HL survivors and HC. Pulse rate tended to be higher in the HL group (*P* = 0.054). The baseline FBF was lower in HL survivors than in HC subjects (*P* = 0.006). The HL survivors had peak V̇o_2_ (*P* = 0.013) and peak pulmonary ventilation (*P* = 0.035) significantly lower compared with HC.

**Table 1.** Physical and hemodynamic characteristics in HL and healthy subjects

	Hodgkin Lymphoma	Healthy Control	*P* Value
*n*	18	10	
Physical characteristics			
Sex, male/female	11/7	7/3	0.892
Age, yr	46 ± 3	42 ± 3	0.420
BMI, kg/m^2^	28.6 ± 1.3	25.6 ± 1.4	0.154
Weight, kg	81.4 ± 3.7	72.1 ± 5.4	0.153
Height, cm	169 ± 0.1	167 ± 0.1	0.528
Maximal aerobic capacity			
Peak V̇o_2_, mL/kg/min	26.4 ± 2.0	34.2 ± 2.1	0.013*
Peak PV, L/min	76.5 ± 2.2	89.9 ± 5.3	0.035*
Hemodynamic parameters			
SAP, mmHg	123 ± 5	125 ± 4	0.726
DAP, mmHg	71 ± 2	68 ± 3	0.458
MAP, mmHg	88 ± 3	87 ± 3	0.801
PR, beats/min	82 ± 4	71 ± 3	0.054*
FBF, mL/min/100 mL	2.1 ± 0.1	2.7 ± 0.1	0.006*
Comorbidities			
Hypertension, *n* (%)	5 (29)	0 (0)	0.098
Diabetes, *n* (%)	5 (29)	1 (10)	0.525

Values are means ± SE. HL, Hodgkin lymphoma survivors; BMI, body mass index; V̇o_2_, oxygen consumption; PV, pulmonary ventilation; SAP, systolic arterial pressure; DAP, diastolic arterial pressure; MAP, mean arterial pressure; PR, pulse rate; FBF, forearm blood flow. Values refer to independent *t* test. *P ≤ 0.05.

#### Responses to mental stress.

**Figure 1.**
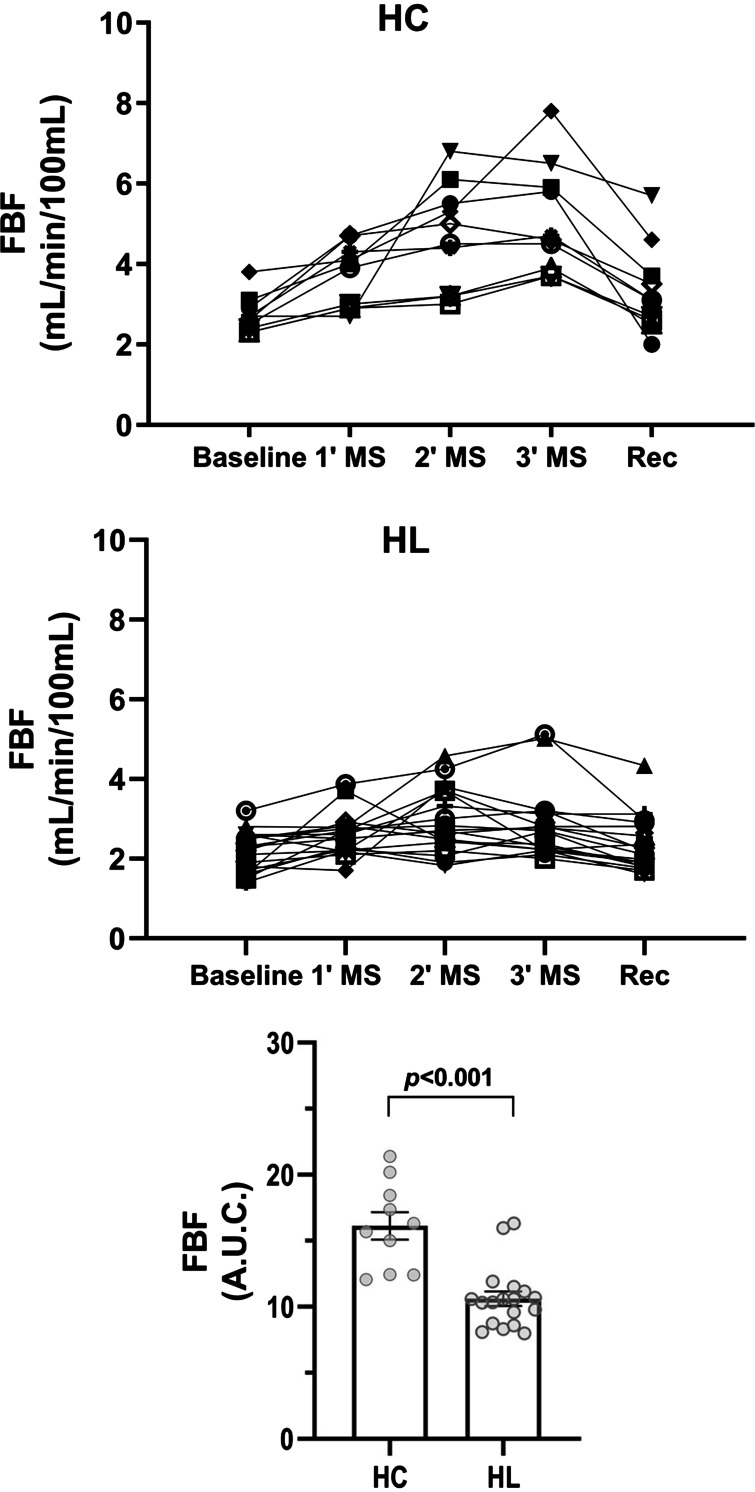
Responses of forearm blood flow during mental stress in Hodgkin lymphoma survivors (HL, *n* = 18) and healthy controls (HC, *n* = 10). Data are represented as means ± SE. AUC, total area under the curve; MS, mental stress; Rec, recovery.

Arterial pressure and pulse rate responses (AUC) throughout the experimental protocol were not different between HL survivors and HC subjects (Supplemental Table S1; all Supplemental Material is available at https://doi.org/10.6084/m9.figshare.25583517). FBF increased during mental stress in both HL survivors and HC. However, the increase in FBF was significantly lower in the HL survivors (AUC, Fig. 1; *P* ≤ 0.001).

### Effects of Exercise Training

#### Preexercise training.

**Figure 2.**
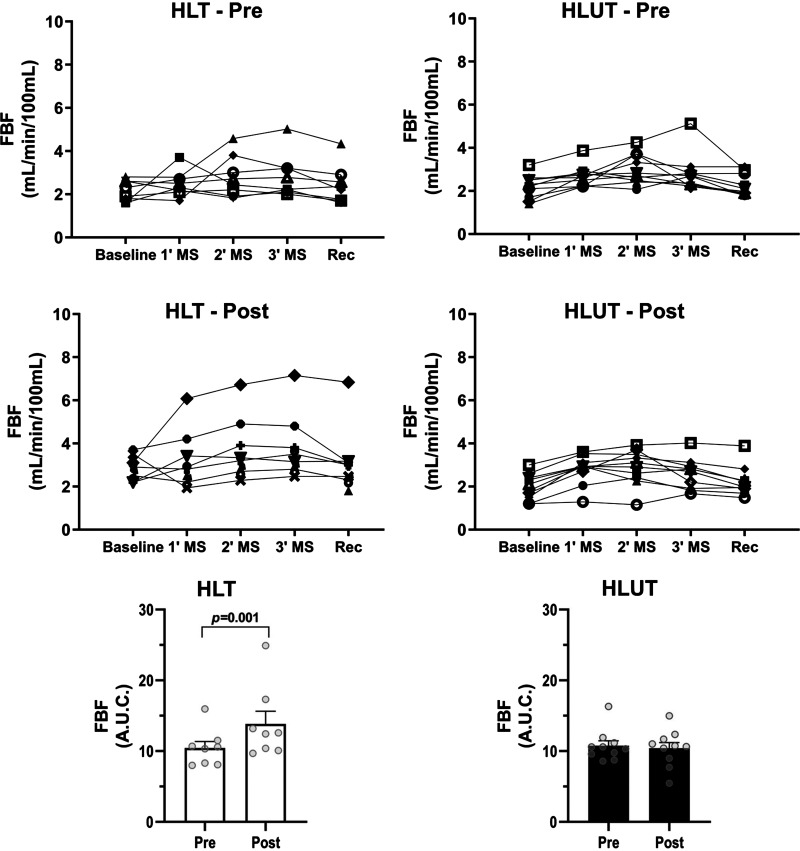
Responses of forearm blood flow during mental stress in exercise-trained Hodgkin lymphoma survivors (HLT, *n* = 8) and untrained survivors (HLUT, *n* = 10). Note the effect of exercise training of increasing forearm blood flow responses (*P* < 0.05). Data are represented as means ± SE. AUC, total area under the curve; MS, mental stress; Rec, recovery.

To test the hypothesis that ET restores muscle blood flow in HL survivors, 18 HL survivors from the initial study plus two additional survivors were randomly divided (1:1 ratio) into two groups: exercise-trained (HLT, *n* = 10) and untrained (HLUT, *n* = 10). Two patients in the HLT group withdrew during the study. Thus, eight patients in the HLT and 10 patients in the HLUT finished the study. There were no differences in baseline physical characteristics, maximal aerobic capacity, and hemodynamic parameters between HLT and HLUT survivors (Table 2). During mental stress, arterial pressure and pulse rate were not different between groups (Supplemental Table S2). Likewise, FBF was not different between groups (Fig. 2).

**Table 2.** Physical and hemodynamic characteristics in HLT and HLUT

	HLT		HLUT		
	Pre	Post	Pre	Post	Interaction *P* Value
*n*	8		10		
Physical characteristics					
Age, yr	50 ± 5	50 ± 5	43 ± 4	43 ± 4	0.277
Sex, male/female	6/2	6/2	5/5	5/5	0.549
BMI, kg/m^2^	26.6 ± 1.8	26.0 ± 1.9†	28.6 ± 1.5	28.7 ± 1.6	0.044
Weight, kg	77.7 ± 5.8	76.0 ± 6.0†	81.8 ± 4.9	81.9 ± 5.0	0.037
Height, cm	170 ± 0.1	170 ± 0.1	169 ± 0.1	169 ± 0.1	0.898
Maximal aerobic capacity					
V̇o_2_ peak, mL/kg/min	30.1 ± 3.2	33.5 ± 3.3*†	24.6 ± 2.7	24.3 ± 2.8	0.001
Peak PV, L/min	79.6 ± 2.7	95.5 ± 3.2*†	73.1 ± 3.1	71.5 ± 3.7	0.002
Hemodynamic parameters					
SAP, mmHg	121 ± 8	117 ± 8	114 ± 9	128 ± 9†	0.026
DAP, mmHg	68 ± 5	66 ± 5	70 ± 6	70 ± 5	0.769
MAP, mmHg	86 ± 6	83 ± 5	84 ± 6	89 ± 6	0.071
PR, beats/min	75 ± 6	68 ± 6	85 ± 5	86 ± 5	0.074
FBF, mL/min/100 mL	2.1 ± 0.2	2.9 ± 0.2*†	2.2 ± 0.2	2.0 ± 0.2	<0.001

Values are means ± SE. BMI, body mass index; V̇o_2_, oxygen consumption; PV, pulmonary ventilation; SAP, systolic arterial pressure; DAP, diastolic arterial pressure; MAP, mean arterial pressure; PR, pulse rate; FBF, forearm blood flow; HLT, exercise-trained Hodgkin lymphoma survivors; HLUT, untrained Hodgkin lymphoma survivors. Values refer to two-way analysis of variance with repeated measures or χ^2^ test. *P < 0.05, between groups comparison; †*P* < 0.05, within groups comparison.

#### Postexercise training.

The attendance at exercise training was very good, ranging minimal of 78% and maximal of 95%. The postexercise training physical characteristics, maximal aerobic capacity, and hemodynamic parameters are shown in Table 2. Body weight (interaction *P* = 0.037, post hoc *P* = 0.016) and body mass index (interaction *P* = 0.044, post hoc *P* = 0.020) were decreased in the HLT survivors. No changes in body weight and body weight index were observed in the HLUT (post hoc *P* = 0.740 and post hoc *P* = 0.742, respectively). Peak V̇o_2_ was increased in the HLT group (interaction *P* < 0.001, post hoc *P* < 0.001). No significant changes were observed in the HLUT group (post hoc *P* = 0.594). Likewise, peak pulmonary ventilation was increased in the HLT group (Interaction *P* = 0.002, post hoc *P* = 0.001). No changes were observed in HLUT (post hoc *P* = 0.525).

No changes in arterial pressure and pulse rate were observed in HLT and HLUT survivors (Table 2). FBF was increased in the HLT survivors (Interaction *P* < 0.001, post hoc *P* < 0.001). In contrast, no changes were found in the HLUT survivors (post hoc, *P* = 0.121).

Arterial pressure and pulse rate levels during mental stress were not changed in the HLT survivors and HLUT survivors (Supplemental Table S2). FBF responses during mental stress were increased in the HLT group (Fig. 2; interaction *P* = 0.004, post hoc *P* = 0.001). No change was observed in the HLUT group (post hoc *P* = 0.643).

Further analysis showed a significant correlation between the changes in peak V̇o_2_ and the changes in FBF (Supplemental Fig. S1, r = 0.711, *P* = 0.001).

## DISCUSSION

The main and new findings of the present study are that long-term HL survivors have blunted FBF responses during mental stress. ET increases FBF during mental stress in long-term HL survivors. These responses support the notion that ET improves muscle blood flow during mental challenges and, in consequence, hemodynamic responses in patients with HL. This is an important finding given that cardiovascular disease is one of the major causes of death in patients with HL (3).

Impairment in muscle blood flow responses during mental stress has been documented in patients with heart failure and, more recently, in COVID-19 survivors (13, 17, 18). The present study extends this knowledge to HL survivors. These survivors have lower muscle blood flow responses during mental stress than in healthy individuals. This new finding has clinical implications. The diminished muscle vasodilation during such a common physiological behavior (mental challenge) may increase even further the cardiovascular risk in HL survivors in whom the medications to treat cancer and the comorbidities associated with lifestyle predispose cardiovascular disease. Our study does not answer these questions, but it certainly opens a new area of investigation in HL survivors.

The mechanisms underlying the lowered muscle blood flow responses during mental stress in HL survivors are out of the scope of our study. However, as muscle vasodilation is governed by the equilibrium between vasodilatory forces and vasoconstrictor forces, someone can propose that the diminished FBF responses in the HL survivors are due to a reduction in endothelial function or an increase in sympathetic nerve activity.

The present study is the first demonstration that ET restores muscle blood flow during mental stress in HL survivors. This finding is suggestive of both amelioration in the circulatory response and reduction in the risk of cardiovascular events during acute stress in this set of patients.

The improvement in muscle blood flow during mental stress may be associated with amelioration in the endothelial function. Some investigators elegantly demonstrated that ET improves endothelium-dependent vasodilation in epicardial coronary vessels as well as in resistance vessels in patients with coronary artery disease (10). In patients with heart failure, ET significantly increases peripheral blood flow in response to intra-arterial infusion of acetylcholine, which demonstrates that ET increases endothelium-mediated skeletal muscle vasodilation (20). It is also possible that the increase in muscle blood flow is due to reduction in sympathetic nerve activity. ET remarkably decreases muscle sympathetic nerve activity in patients with heart failure (14), regardless of sex and age (21, 22). Of course, we cannot rule out that an increase in muscle size and capillarization contributed to the improvement in muscle blood flow (23). Unfortunately, there is no information regarding the muscle force in our study. This is an interesting topic for future investigations.

We observed that ET increases cardiorespiratory fitness in patients with HL. This observation is in line with previous studies. Oldervoll and collaborators (24) reported that home-based exercise program improved maximal aerobic capacity in HL survivors. Courneya and collaborators (25) described that supervised aerobic ET increased maximal aerobic capacity in patients with HL or non-HL who received chemotherapy or no treatments. The novelty in our study is the correlation between the changes in FBF responses during mental stress and the changes in peak V̇o_2_. What is the physiological relevance of this finding? As the increase in FBF during mental stress is an indicative of amelioration in muscle blood flow, someone can suggest that the improvement in muscle blood flow responses favors the increase in maximal aerobic capacity in HL survivors.

Someone could argue that the withdrawal of two patients in the HLT group limits our study. This is unlikely because our results are consistent and sufficient to show the effects of ET in HL survivors.

### Perspectives and Significance

Stroop Color Word test is an adequate and practical strategy to examine muscle blood flow alteration in long-term HL survivors. Thus, this test may be useful in the clinical practice of patients with HL. Rehabilitation programs during and after treatment in patients with HL have been a matter of investigation. However, there is not enough evidence for the recommendation of rehabilitation for HL survivors (26). The exercise training models are quite variable, which limits adequate interpretation of the physiological responses. The present study shows that supervised moderate aerobic exercise training in combination with local strength exercises for 4 mo increases muscle blood flow during mental challenge in long-term HL survivors. In addition, our exercise paradigm significantly increases maximal aerobic capacity. These findings provide evidence for the safety and effectiveness of exercise training in the treatment of patients with HL.

### Conclusion

In conclusion, HL survivors have impaired MBF responses during mental challenge. ET restores muscle blood flow at rest and during mental stress in HL survivors. These findings suggest that ET improves vascular function and hemodynamic responses during stressful states in this set of survivors. The association between the gain in maximal aerobic capacity and the increase in muscle blood flow responses during mental stress supports the notion that an improvement in muscle vascular function supports an increase in maximal aerobic capacity in HL survivors.

## DATA AVAILABILITY

Data will be made available upon reasonable request to the corresponding author.

## SUPPLEMENTAL DATA

10.6084/m9.figshare.25583517 Supplemental Tables S1 and S2 and Supplemental Fig. S1: https://doi.org/10.6084/m9.figshare.25583517.

## GRANTS

This study was supported by Fundação de Amparo à Pesquisa do Estado de São Paulo (FAPESP) Grant 2015/22814-5. C.E.N. is supported by Conselho Nacional de Desenvolvimento Científico e Tecnológico (CNPq) Grant 304697/2020-6. B.R.G-S. is supported by Coordenação de Aperfeiçoamento de Pessoal de Nível Superior (CAPES) Grant 88887.829284/2023-00. E.T.D. is supported by FAPESP Grants 2020/03375-9 and 2021/03076-4 and CNPq Grant 307434/2021-4.

## DISCLOSURES

M.V.N. reports receiving research funding to institution from Mirati, Novartis, Checkmate, Alaunos, AstraZeneca, Pfizer, Genentech, Navire; a consultant or advisory role for Mirati, Merck/MSD, Novartis, Genentech, Sanofi; and other support from Ziopharm Oncology, ApotheCom, Ashfield Healthcare. M.H.H.S.R. reports being senior medical advisor for pharma vigilance and senior medical director for global patient safety for Eli Lilly Brazil. None of the other authors has any conflicts of interest, financial or otherwise, to disclose.

## AUTHOR CONTRIBUTIONS

L.d.S.S., M.H.H.d.S.R., M.V.N., L.A.H., R.K.F., and C.E.N. conceived and designed research; L.d.S.S., C.J.P., U.U., and N.S.G. performed experiments; L.d.S.S., B.R.G-S., E.T.D., C.J.P., and C.E.N. analyzed data; L.d.S.S., B.R.G-S., and C.E.N. interpreted results of experiments; L.d.S.S. and B.R.G-S. prepared figures; L.d.S.S., B.R.G-S., and C.E.N. drafted manuscript; L.d.S.S., and B.R.G-S., and C.E.N. edited and revised manuscript; L.d.S.S., M.H.H.d.S.R., M.V.N., B.R.G-S., E.T.D., C.J.P., U.U., N.S.G., L.A.H., and R.K.F., C.E.N. approved final version of manuscript.

## References

[1] Brice P, de Kerviler E, Friedberg JW. Classical Hodgkin lymphoma. Lancet 398: 1518–1527, 2021. doi:10.1016/S0140-6736(20)32207-8. 33493434

[2] Hayek SS, Ganatra S, Lenneman C, Scherrer-Crosbie M, Leja M, Lenihan DJ, Yang E, Ryan TD, Liu J, Carver J, Mousavi N, O'Quinn R, Arnold A, Banchs J, Barac A, Ky B. Preparing the cardiovascular workforce to care for oncology patients: JACC Review Topic of the Week. J Am Coll Cardiol 73: 2226–2235, 2019. doi:10.1016/j.jacc.2019.02.041. 31047011 PMC7153911

[3] Lu Z, Teng Y, Ning X, Wang H, Feng W, Ou C. Long-term risk of cardiovascular disease mortality among classic Hodgkin lymphoma survivors. Cancer 128: 3330–3339, 2022. doi:10.1002/cncr.34375. 35872619

[4] Daskalaki M, Makris T, Vassilakopoulos T, Moyssakis I, Siakantaris M, Angelopoulou M, Papadogiannis D, Vaiopoulos G, Pangalis G. Effects of anthracyclines on aortic distensibility in patients with lymphomas: a prospective study. Hellenic J Cardiol 55: 191–196, 2014. 24862610

[5] Zelcer S, Chen B, Mangel J, Vujovic O, Thiessen-Philbrook HR, Reider M, Mahmud FH. Impaired vascular function in asymptomatic young adult survivors of Hodgkin lymphoma following mediastinal radiation. J Cancer Surviv 4: 218–224, 2010. doi:10.1007/s11764-010-0138-6. 20652436

[6] Abouzid MRA, Hameed M, Katta MR, Valisekka SS. Approach to lymphoma-associated cardiomyopathy. Cardiol Rev 32: 104–109, 2024. doi:10.1097/CRD.0000000000000471. 36129332

[7] Lenneman CG, Sawyer DB. Cardio-oncology: an update on cardiotoxicity of cancer-related treatment. Circ Res 118: 1008–1020, 2016. doi:10.1161/CIRCRESAHA.115.303633. 26987914

[8] Yu AF, Jones LW. Modulation of cardiovascular toxicity in Hodgkin lymphoma: potential role and mechanisms of aerobic training. Future Cardiol 11: 441–452, 2015. doi:10.2217/fca.15.29. 26234325 PMC5558532

[9] Gilchrist SC, Barac A, Ades PA, Alfano CM, Franklin BA, Jones LW, La Gerche A, Ligibel JA, Lopez G, Madan K, Oeffinger KC, Salamone J, Scott JM, Squires RW, Thomas RJ, Treat-Jacobson DJ, Wright JS; American Heart Association Exercise, Cardiac Rehabilitation, and Secondary Prevention Committee of the Council on Clinical Cardiology; Council on Cardiovascular and Stroke Nursing; Council on Peripheral Vascular Disease. Cardio-oncology rehabilitation to manage cardiovascular outcomes in cancer patients and survivors: a scientific statement from the American Heart Association. Circulation 139: e997–e1012, 2019. doi:10.1161/CIR.0000000000000679. 30955352 PMC7603804

[10] Hambrecht R, Wolf A, Gielen S, Linke A, Hofer J, Erbs S, Schoene N, Schuler G. Effect of exercise on coronary endothelial function in patients with coronary artery disease. N Engl J Med 342: 454–460, 2000. doi:10.1056/NEJM200002173420702. 10675425

[11] Laufs U, Werner N, Link A, Endres M, Wassmann S, Jurgens K, Miche E, Bohm M, Nickenig G. Physical training increases endothelial progenitor cells, inhibits neointima formation, and enhances angiogenesis. Circulation 109: 220–226, 2004. doi:10.1161/01.CIR.0000109141.48980.37. 14691039

[12] Trevizan PF, Antunes-Correa LM, Lobo DML, Oliveira PA, de Almeida DR, Abduch MCD, Mathias Junior W, Hajjar LA, Kalil Filho R, Negrao CE. Effects of inspiratory muscle training combined with aerobic exercise training on neurovascular control in chronic heart failure patients. ESC Heart Fail 8: 3845–3854, 2021. doi:10.1002/ehf2.13478. 34184426 PMC8497326

[13] Middlekauff HR, Nguyen AH, Negrao CE, Nitzsche EU, Hoh CK, Natterson BA, Hamilton MA, Fonarow GC, Hage A, Moriguchi JD. Impact of acute mental stress on sympathetic nerve activity and regional blood flow in advanced heart failure: implications for 'triggering' adverse cardiac events. Circulation 96: 1835–1842, 1997. doi:10.1161/01.cir.96.6.1835. 9323069

[14] Roveda F, Middlekauff HR, Rondon MU, Reis SF, Souza M, Nastari L, Barretto AC, Krieger EM, Negrao CE. The effects of exercise training on sympathetic neural activation in advanced heart failure: a randomized controlled trial. J Am Coll Cardiol 42: 854–860, 2003. doi:10.1016/s0735-1097(03)00831-3. 12957432

[15] Lang RM, Badano LP, Mor-Avi V, Afilalo J, Armstrong A, Ernande L, Flachskampf FA, Foster E, Goldstein SA, Kuznetsova T, Lancellotti P, Muraru D, Picard MH, Rietzschel ER, Rudski L, Spencer KT, Tsang W, Voigt J-U. Recommendations for cardiac chamber quantification by echocardiography in adults: an update from the American Society of Echocardiography and the European Association of Cardiovascular Imaging. J Am Soc Echocardiogr 28: 1–39.e14, 2015. doi:10.1016/j.echo.2014.10.003. 25559473

[16] Antunes-Correa LM, Nobre TS, Groehs RV, Alves MJ, Fernandes T, Couto GK, Rondon MU, Oliveira P, Lima M, Mathias W, Brum PC, Mady C, Almeida DR, Rossoni LV, Oliveira EM, Middlekauff HR, Negrao CE. Molecular basis for the improvement in muscle metaboreflex and mechanoreflex control in exercise-trained humans with chronic heart failure. Am J Physiol Heart Circ Physiol 307: H1655–H1666, 2014. doi:10.1152/ajpheart.00136.2014. 25305179 PMC4255006

[17] Negrao CE, Hamilton MA, Fonarow GC, Hage A, Moriguchi JD, Middlekauff HR. Impaired endothelium-mediated vasodilation is not the principal cause of vasoconstriction in heart failure. Am J Physiol Heart Circ Physiol 278: H168–H174, 2000. doi:10.1152/ajpheart.2000.278.1.H168. 10644596

[18] Faria D, Moll-Bernardes R, Testa L, Moniz CMV, Rodrigues EC, Mota JM, Souza FR, Alves M, Ono BE, Izaias JE, Sales AO, Rodrigues TS, Salemi VMC, Jordao CP, De Angelis K, Craighead DH, Rossman MJ, Bortolotto LA, Consolim-Colombo FM, Irigoyen MC-C, Seals DR, Negrao CE, Sales ARK. Neurovascular and hemodynamic responses to mental stress and exercise in severe COVID-19 survivors. Am J Physiol Regul Integr Comp Physiol 325: R269–R279, 2023. doi:10.1152/ajpregu.00111.2023. 37449870 PMC10625836

[19] Stenehjem JS, Smeland KB, Murbraech K, Holte H, Kvaloy S, Thorsen L, Arbo I, Jones LW, Aakhus S, Lund MB, Kiserud CE. Cardiorespiratory fitness in long-term lymphoma survivors after high-dose chemotherapy with autologous stem cell transplantation. Br J Cancer 115: 178–187, 2016. doi:10.1038/bjc.2016.180. 27351215 PMC4947700

[20] Hambrecht R, Fiehn E, Weigl C, Gielen S, Hamann C, Kaiser R, Yu J, Adams V, Niebauer J, Schuler G. Regular physical exercise corrects endothelial dysfunction and improves exercise capacity in patients with chronic heart failure. Circulation 98: 2709–2715, 1998. doi:10.1161/01.cir.98.24.2709. 9851957

[21] Antunes-Correa LM, Kanamura BY, Melo RC, Nobre TS, Ueno LM, Franco FG, Roveda F, Braga AM, Rondon MU, Brum PC, Barretto AC, Middlekauff HR, Negrao CE. Exercise training improves neurovascular control and functional capacity in heart failure patients regardless of age. Eur J Prev Cardiol 19: 822–829, 2012. doi:10.1177/1741826711414626. 21697210

[22] Antunes-Correa LM, Melo RC, Nobre TS, Ueno LM, Franco FG, Braga AM, Rondon MU, Brum PC, Barretto AC, Middlekauff HR, Negrao CE. Impact of gender on benefits of exercise training on sympathetic nerve activity and muscle blood flow in heart failure. Eur J Heart Fail 12: 58–65, 2010. doi:10.1093/eurjhf/hfp168. 20023046 PMC2796143

[23] Traylor MK, Bauman AJ, Saiyasit N, Frizell CA, Hill BD, Nelson AR, Keller JL. An examination of the relationship among plasma brain derived neurotropic factor, peripheral vascular function, and body composition with cognition in midlife African Americans/Black individuals. Front Aging Neurosci 14: 980561, 2022. doi:10.3389/fnagi.2022.980561. 36092801 PMC9453229

[24] Oldervoll LM, Kaasa S, Knobel H, Loge JH. Exercise reduces fatigue in chronic fatigued Hodgkins disease survivors–results from a pilot study. Eur J Cancer 39: 57–63, 2003. doi:10.1016/s0959-8049(02)00483-5. 12504659

[25] Courneya KS, Sellar CM, Stevinson C, McNeely ML, Peddle CJ, Friedenreich CM, Tankel K, Basi S, Chua N, Mazurek A, Reiman T. Randomized controlled trial of the effects of aerobic exercise on physical functioning and quality of life in lymphoma patients. J Clin Oncol 27: 4605–4612, 2009. doi:10.1200/JCO.2008.20.0634. 19687337

[26] Amatya B, Khan F, Lew TE, Dickinson M. Rehabilitation in patients with lymphoma: an overview of Systematic Reviews. J Rehabil Med 53: jrm00163, 2021. doi:10.2340/16501977-2810. 33710351 PMC8814843

